# A New Model of Hemoglobin Oxygenation

**DOI:** 10.3390/e24091214

**Published:** 2022-08-30

**Authors:** Igor A. Lavrinenko, Gennady A. Vashanov, José L. Hernández Cáceres, Anatoly S. Buchelnikov, Yury D. Nechipurenko

**Affiliations:** 1 Department of Human and Animal Physiology, Voronezh State University, Universitetskaya Sq. 1, 394018 Voronezh, Russia; 2 Cuban Neuroscience Center, 15202 Avenida 25, Playa, La Habana 11600, Cuba; 3 Laboratory of Molecular and Cellular Biophysics, Sevastopol State University, Universitetskaya Str. 33, 299053 Sevastopol, Russia; 4 Laboratory of DNA-Protein Interactions, Engelhardt Institute of Molecular Biology, Russian Academy of Sciences, Vavilova Str. 32, 119991 Moscow, Russia

**Keywords:** cooperative binding of ligands, oxyhemoglobin dissociation curve, Hill equation, Hill coefficient, allosteric interactions

## Abstract

The study of hemoglobin oxygenation, starting from the classical works of Hill, has laid the foundation for molecular biophysics. The cooperative nature of oxygen binding to hemoglobin has been variously described in different models. In the Adair model, which better fits the experimental data, the constants of oxygen binding at various stages differ. However, the physical meaning of the parameters in this model remains unclear. In this work, we applied Hill’s approach, extending its interpretation; we obtained a good agreement between the theory and the experiment. The equation in which the Hill coefficient is modulated by the Lorentz distribution for oxygen partial pressure approximates the experimental data better than not only the classical Hill equation, but also the Adair equation.

## 1. Introduction

Cooperativity in allosteric systems such as oligomeric proteins [1,2] provides the ability to rapidly regulate their functional activity. Recent work has shown that allosteric interactions expand the ability of biological systems to store information [3]. It has also been demonstrated that cooperative interactions lead to an entropy reduction in the system [4]. In particular, the cooperation between the subunits of ion channels during nerve impulse generation potentiates the ability of neurons and nerve networks to process information [5].

In this connection, the study of cooperative interactions is of general biological importance [6,7,8]. Of particular relevance is the study of the cooperativity of hemoglobin oxygenation [9]. This macromolecule is a convenient model for studying the mechanisms of the enzymatic reactions of oligomers because its active center is similar to the catalytic center of the enzyme [10]. The ability of hemoglobin to hold a ligand in the active center for a long time makes it possible to study its structural state; e.g., the short-term state of the enzyme–substrate complex [11,12]. This probably played a role in the choice of hemoglobin molecule by M. Perutz to study the spatial structure of complex proteins [13].

Over 100 years ago, Hill proposed an empirical equation to describe the hemoglobin oxygenation curve, which has found an application not only in describing the sigmoid shape of the oxygenation curve, but also in a number of other fields from enzymology [14,15], toxicology [16] and pharmacology [17] to problems of modeling gene transcription regulation [18,19] as well as in models describing potential-dependent ion channel transitions [20,21] among others.

At the same time, Hill’s approach may be more than just a descriptive theory characterizing the cooperative effect. The equation demonstrates interesting explanatory and mechanistic properties in direct connection with the law of mass action [22,23,24].

Previously, we proposed a mathematical model for hemoglobin oxygenation [25,26] and ascertained that our model fits better to experimental oxyhemoglobin dissociation curves when compared with the classical Hill equation and is comparable with the Adair equation (Adair–Klotz) [26]. However, for oxygen partial pressure levels below 3 mm Hg, the proposed equation is inferior to the Adair equation in accuracy approximation.

Thus, considering the importance of evaluating the cooperative interactions in the macromolecule at the initial stage of its oxygenation, we further analyzed the previously proposed equation and searched for a more accurate approximating model.

## 2. Materials and Methods

The subjects of this study were the Hill model and a set of experimental data obtained by Winslow et al. [27] and Severinghaus [28]. The optimization of the model parameters was performed by the generalized reduced gradient (GRG) method [29], where the target function was the sum of least squares (LS) method [30]. The evaluation of the degree of model fit to the experimental data was performed through the coefficient of determination [31]. Confidence and prediction bands for the studied non-linear models were calculated at α = 0.0001 [32]. The necessary calculations were performed in MS Excel.

## 3. Results

### 3.1. The Hill Equation and Its Limitations

The Hill equation, in general terms, assumes the simultaneous binding of ligand molecules by all oligomer subunits:

(1) $$y=\frac{p^{h}}{p_{50}^{h}+p^{h}}$$

where, in the case of oxygenation, *y* is the degree of saturation of hemoglobin by oxygen, *p* is the partial pressure of O_2_, *p*_50_ is the oxygen pressure at which half of the macromolecules are saturated by the ligand and *h* is the Hill coefficient.

Currently, this equation, which can formally be deduced from the law of mass action, is viewed primarily as a formal description of the interaction of ligands with the receptor sites of the protein and is defined by the corresponding microscopic constants of the equilibrium reactions. Their product is a macroscopic constant equal to (*p*_50_)^−*h*^. However, it is not possible to correlate the values of these microscopic constants in the Hill equation. For the hemoglobin tetramer, this expression can be represented as follows:

(2) $$y=\frac{k_{1}p\cdot k_{2}p\cdot k_{3}p\cdot k_{4}p}{1+k_{1}p\cdot k_{2}p\cdot k_{3}p\cdot k_{4}p}=\frac{k_{1}\cdot k_{2}\cdot k_{3}\cdot k_{4}p^{4}}{1+k_{1}\cdot k_{2}\cdot k_{3}\cdot k_{4}p^{4}}=\frac{k^{4}p^{4}}{1+k^{4}p^{4}}=\frac{Kp^{4}}{1+Kp^{4}}=\frac{p^{4}}{p_{50}^{4}+p^{4}},$$

where *k*_1_, *k*_2_, *k*_3_ and *k*_4_ are the equilibrium reaction constants for each of the subunits; *k*^4^ is the product of constants *k*_1_–*k*_4_; and *K* is the macroscopic constant.

A similar situation arises when analyzing the Adair (Adair–Klotz) equation, which is a development of the Hill equation, considering the product of these microscopic constants for each of the oxygenation stages:

(3) $$y=\frac{k_{1}p+2\cdot k_{1}k_{2}p^{2}+3\cdot k_{1}k_{2}k_{3}p^{3}+4\cdot k_{1}k_{2}k_{3}k_{4}p^{4}}{4\cdot \left(1+k_{1}p+k_{1}k_{2}p^{2}+k_{1}k_{2}k_{3}p^{3}+k_{1}k_{2}k_{3}k_{4}p^{4}\right)}=\frac{K_{1}p+2K_{2}p^{2}+3K_{3}p^{3}+4K_{4}p^{4}}{4\left(1+K_{1}p+K_{2}p^{2}+K_{3}p^{3}+K_{4}p^{4}\right)}.$$

However, in the Adair–Klotz equation, the microscopic constants can be determined from the corresponding calculated values of the macroscopic constants. It should also be noted that the equilibrium reaction constants of the above equation are apparent and it is not clear from them what the value of *p*_50_ is [33]. As this equation consists of four components and is given by the same number of fitting coefficients (*K*_1_–*K*_4_), it is expected to have a higher approximation capability relative to the Hill and Bernard equations [25].

For the Hill equation describing hemoglobin oxygenation, there is a pronounced discrepancy between the theoretical value of the degree index *h*, corresponding with the number of subunits, and the calculated value *h*, which makes it difficult to understand the physical meaning of this value [26,34,35,36].

This equation is also characterized by a poor approximation of the experimental data at the calculated value of *h* in the range of the partial oxygen pressures where the degree of saturation of hemoglobin with the ligand is outside the range of 20–80%, especially for low *p*-values. This circumstance indirectly points to the variability of the Hill coefficient during oxygen binding by hemoglobin [37].

### 3.2. Phenomenological Model of Oligomer Liganding

We proposed a hypothetical model of ligand binding by the subunits of a macromolecule, which was defined by the following conditions:

1. Each subunit of the oligomer could be either vacant for the ligand or liganded and also in different conformational states;
2. In these conformational states, the oligomer subunits experienced coordinated perturbation by neighboring subunits;
3. The current superposition of the conformational states of the subunit states could change the affinity of a subunit for an attachable or already attached ligand;
4. Ligand binding reconfigured the interaction between the subunits and created a new self-consistent level of cooperativity between them, which determined the nature and strength of the interaction of the attachable or already attached ligand according to a feedback mechanism.

**Figure 1:**
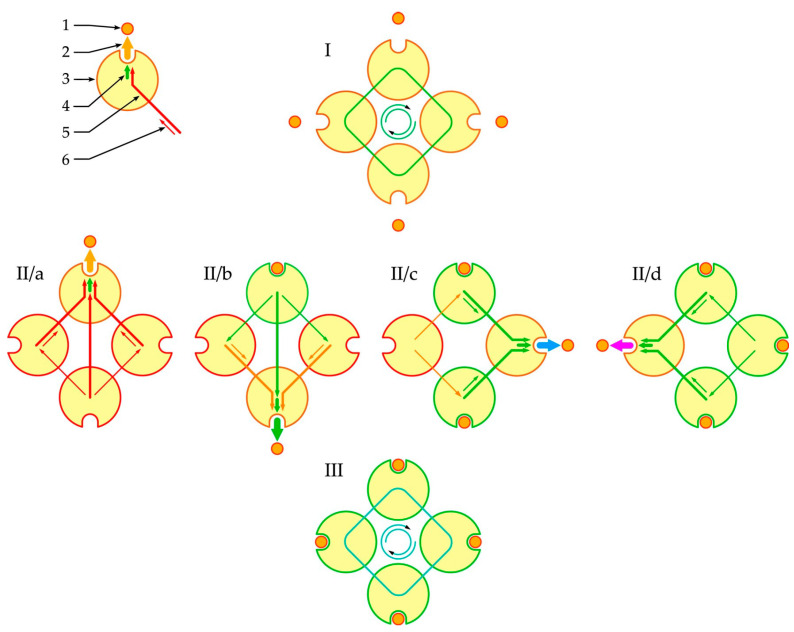
Phenomenological model of oligomer liganding. I, initial (unliganded) state of oligomer; II/a–d, liganding steps (hemoglobin oxygenation); III, terminal state (complete liganding). Digits indicate: 1, ligand; 2, resultant interaction of oligomer with ligand; 3, oligomer; 4, interaction component of the liganded subunit; 5, interaction component of the conjugated subunit; 6, mediated interaction component through the conjugated subunit. Color of arrows and subunits denotes from inhibition (warm) to promotion (cold) of oxygenation.

Thus, based on this model (Figure 1), the Hill coefficient could be viewed as a function of the number of interacting subunits and the degree of cooperativity between them modulated by the degree of the vacancy filling with the corresponding ligand.

### 3.3. The Hill Coefficient as a Function of Oxygen Partial Pressure

Based on the proposed model (Figure 1), we suggested considering the Hill coefficient as a function of the partial pressure of oxygen conjugated with the given number of interacting subunits. The dependence of this coefficient upon the partial pressure of oxygen was demonstrated in classical works [37,38] by calculating *h* using the known values of the degree of hemoglobin oxygen saturation, the partial pressure of O_2_ and the value of *p*_50_. However, a function allowing a fit with the empirical oxygenation curve has not yet been proposed [38].

Earlier [26], we attempted to develop a new mathematical model of hemoglobin oxygenation. This model has a better approximation of experimental data on hemoglobin oxygenation relative to Hill’s equation and is comparable with Adair’s equation; however, it describes hemoglobin oxygenation at its initial stages worse than the latter. It should also be noted that there are several publications in which macromolecule-binding constants are also considered as functions of macromolecule filling by ligands [25,39,40,41].

We presented the conclusion and justified the equations that were used to calculate the Hill coefficient as a function of the partial pressure of oxygen.

For the original Hill equation, the value of *h* is independent of the partial pressure of oxygen *pO*_2_ and appears as:

(4) $$h=h_{i}=h_{\max}=\bar{h}=\mathrm{const},$$

where *h*_max_ is the maximum possible value of the Hill coefficient in a given interval of *pO*_2_, *h_i_* is the Hill coefficient at a certain *pO*_2_ and $\bar{h}$ is its average value.

Assuming that *h_i_* cannot be greater than *h*_max_, but depends on *p_i_*, the dependence of *h_i_* on *p_i_* (*p_i_* > 1) can be represented by the relation:

(5) $$h_{i}=\frac{h_{\max}}{p_{i}},$$

where *p_i_* is the partial pressure of oxygen at the *i*-th point of the measurement.

For *h_i_*, we could find the value of *p_i_* at which *h_i_* = *h*_max_, with *p_i_* − *p*_max_ = 1 (the value of the partial pressure of oxygen at which *h*_max_ could be detected; the parameter *p*_max_ ≥ 0 shifted the function plot along the horizontal axis):

(6) $$h_{i}=\frac{h_{\max}}{p_{i}-p_{\max}}.$$

The value of *h_i_* must be greater than 0 for any *p_i_* − *p*_max_. Equation (6) would then be represented as the ratio of *h*_max_ to the square of the difference of *p_i_* and *p*_max_:

(7) $$h_{i}=\frac{h_{\max}}{{\left(p_{i}-p_{\max}\right)}^{2}}.$$

In the range of values where (*p_i_* − *p*_max_)^2^ belongs to an open interval from 0 to 1, the value *h_i_* > *h*_max_ and is undefined when the square of this difference is zero.

Representing (*p_i_* − *p*_max_)^2^ as the exponent on any basis greater than unity, Equation (7) could then be presented as follows, where (*p_i_* − *p*_max_)^2^ = 0 and *h_i_* = *h*_max_:

(8) $$h_{i}=\frac{h_{\max}}{\exp{\left(p_{i}-p_{\max}\right)}^{2}}.$$

Using an arbitrary factor *a* to (*p_i_* − *p*_max_)^2^ only scales the argument and does not eliminate the discontinuity of the presented function at the zero point for a given difference:

(9) $$h_{i}=\frac{h_{\max}}{a{\left(p_{i}-p_{\max}\right)}^{2}}.$$

However, by representing the denominator as a sum, we could also obtain *h_i_* = *h*_max_ at (*p_i_* − *p*_max_)^2^ = 0 in the same way as Equation (8):

(10) $$h_{i}=\frac{h_{\max}}{1+{\left(p_{i}-p_{\max}\right)}^{2}}.$$

In Equations (9) and (10), we introduced a scale parameter *s* along the horizontal axis:

(11) $$h_{i}=\frac{h_{\max}}{\exp{\left[\left(p_{i}-p_{\max}\right)/s\right]}^{2}},$$

(12) $$h_{i}=\frac{h_{\max}}{1+{\left[\left(p_{i}-p_{\max}\right)/s\right]}^{2}}.$$

As the experimental dependence of the Hill coefficient on the logarithm of the partial oxygen pressure is bell-shaped, Equations (11) and (12) could be transformed:

(13) $$h_{i}=\frac{h_{\max}}{\exp{\left[\ln\left(p_{i}/p_{\max}\right)/s\right]}^{2}},$$

(14) $$h_{i}=\frac{h_{\max}}{1+{\left[\ln\left(p_{i}/p_{\max}\right)/s\right]}^{2}}.$$

As the Hill coefficient in the Hill equation is equal to one in the absence of cooperativity, Equations (13) and (14) could be reduced to the following form, thus shifting the statistical distribution along the vertical axis by this unity:

(15) $$h_{i\left(-1\right)}=\frac{h_{\max\left(-1\right)}}{\exp{\left[\ln\left(p_{i}/p_{\max}\right)/s\right]}^{2}}+1,$$

(16) $$h_{i\left(-1\right)}=\frac{h_{\max\left(-1\right)}}{1+{\left[\ln\left(p_{i}/p_{\max}\right)/s\right]}^{2}}+1.$$

The substitution of the Hill equation:

(17) $$y_{i}=\frac{p_{i}^{h_{i}}}{p_{50}^{h_{i}}+p_{i}^{h_{i}}},$$

instead of *h_i_* could transform Equation (15) or (16) based on Gauss and Lorentz distributions, respectively, to two models describing hemoglobin oxygenation, where *y_i_* is the degree of ligand saturation at *p_i_*.

Each of the models has four adjustable parameters: *p*_50_; *h*_max(−1)_; ln *p*_max_; and *s* (*s_g_* and *s_l_* are scale parameters for the Gaussian and Lorentz distributions, respectively).

### 3.4. Evaluation of the Effectiveness of Approximation of Experimental Data by Modified Hill Equations

**Figure 2:**
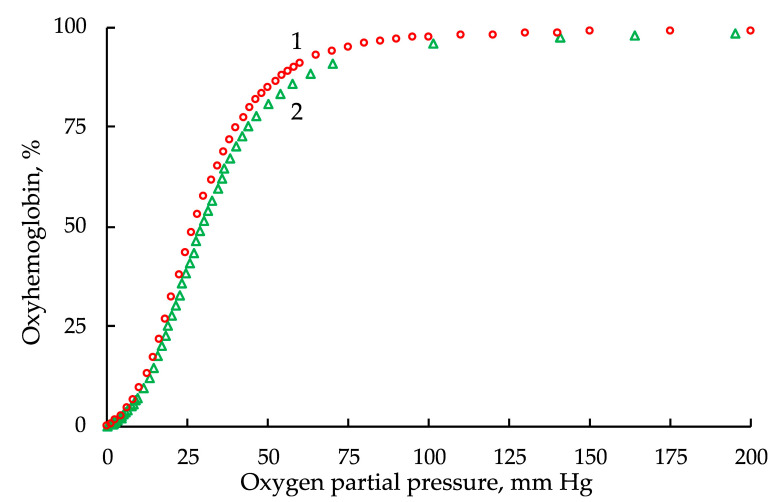
Experimental data points of the hemoglobin dissociation curve: 1, according to Winslow et al. [27] and 2, according to Severinghaus [28].

According to the experimental data of Winslow et al. [27] and Severinghaus [28] (Figure 2), approximating oxyhemoglobin dissociation curves were obtained using the classical (Hill classic) and modified Hill equations with Gauss (Hill/G) and Lorentz (Hill/L) distributions and the Adair equation.

We determined the coefficient of determination (*r*^2^) for the given equations and ranked them in descending order of *r*^2^ (Table 1 and Table 2).

As the tables show, the modified Hill equation with the Lorentz distribution (Hill/L) demonstrated the best approximation results for the two sets of experimental data.

**Figure 3:**
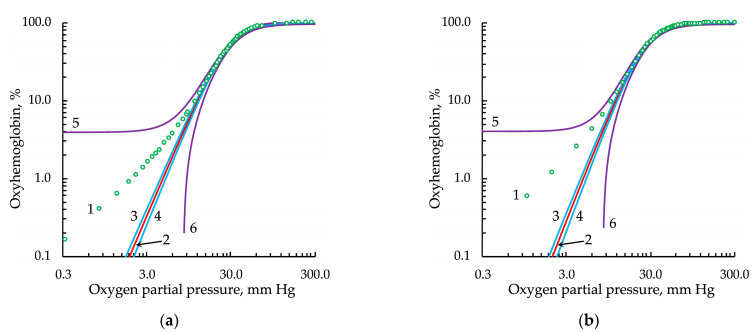
Approximation of the experimental oxygenation curve, represented in logarithmic coordinates, by the classical Hill equation: (**a**) from the data set of Winslow et al. [27] and (**b**) from the data set of Severinghaus [28]. Legend: 1, experimental data points; 2, approximation; 3 and 4, upper and lower bounds of the confidence intervals, respectively; 5 and 6, upper and lower bounds of the prediction intervals, respectively. For all types of intervals, the confidence probability was 0.9999.

**Figure 4:**
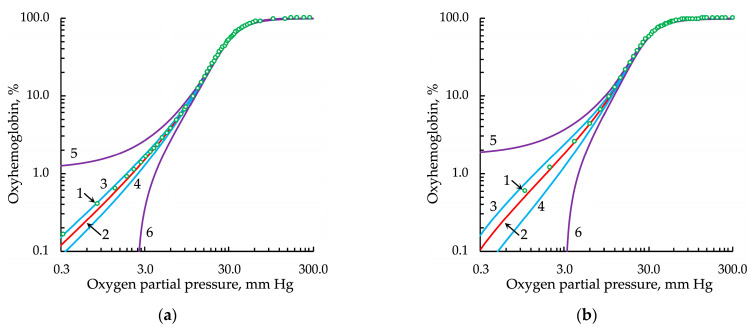
Approximation of the experimental oxygenation curve, represented in logarithmic coordinates, by the Adair equation: (**a**) from the data set of Winslow et al. [27] and (**b**) from the data set of Severinghaus [28]. Legend: 1, experimental data points; 2, approximation; 3 and 4, upper and lower bounds of the confidence intervals, respectively; 5 and 6, upper and lower bounds of the prediction intervals, respectively. For all types of intervals, the confidence probability was 0.9999.

**Figure 5:**
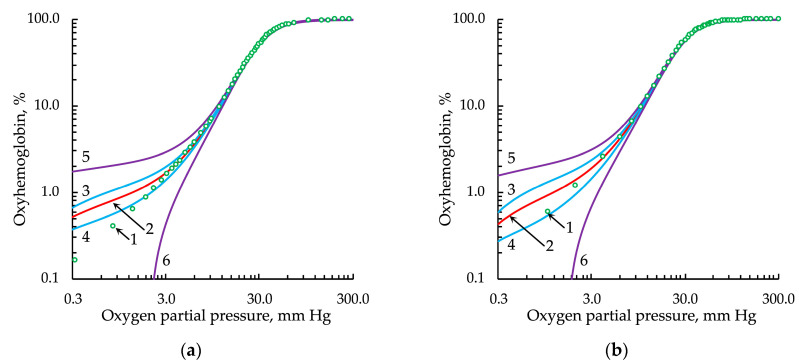
Approximation of the experimental oxygenation curve, represented in logarithmic coordinates, by the Hill equation with *h* modulated by the Gauss distribution: (**a**) from the data set of Winslow et al. [27] and (**b**) from the data set of Severinghaus [28]. Legend: 1, experimental data points; 2, approximation; 3 and 4, upper and lower bounds of the confidence intervals, respectively; 5 and 6, upper and lower bounds of the prediction intervals, respectively. For all types of intervals, the confidence probability was 0.9999.

**Figure 6:**
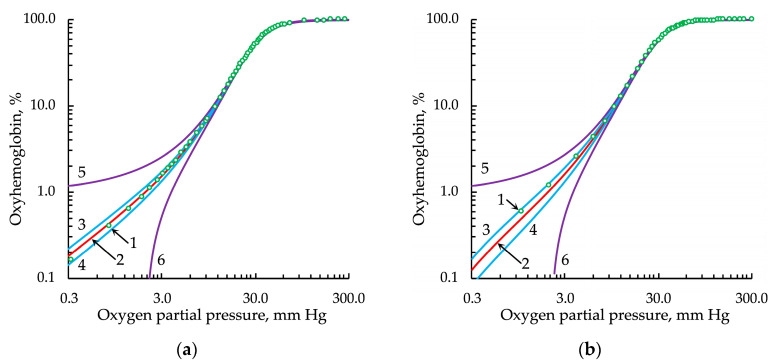
Approximation of the experimental oxygenation curve, represented in logarithmic coordinates, by the Hill equation with *h* modulated by the Lorentz distribution: (**a**) from the data set of Winslow et al. [27] and (**b**) from the data set of Severinghaus [28]. Legend: 1, experimental data points; 2, approximation; 3 and 4, upper and lower bounds of the confidence intervals, respectively; 5 and 6, upper and lower bounds of the prediction intervals, respectively. For all types of intervals, the confidence probability was 0.9999.

As hemoglobin oxygenation in the range of partial gas pressure up to 3 mm Hg was of particular interest in our studies, we analyzed the ODC at low *pO*_2_ values (Figure 3, Figure 4, Figure 5 and Figure 6).

The classical Hill equation, as noted in [42], did not satisfactorily approximate the experimental data in the range of low *pO*_2_ values (Figure 3).

This discrepancy could indicate that, at low oxygen partial pressures, the degree of cooperativity of the subunits in the macromolecule calculated for these values was lower than that predicted within the classical model.

The Adair equation (Figure 4) has four adjustable parameters and relies on the model of sequential ligand binding, which is phenomenologically more reasonable. In this case, at a confidence probability of 0.9999, the experimental points of the curve did not go beyond the confidence interval.

The Hill equation modulated by the Gauss distribution (Hill/G) that we obtained did not satisfactorily approximate the experimental data in the region of low oxygen partial values because of the shape of this distribution conditioned by the exponential dependence (Equation (8); Figure 5).

The application of the Lorentz distribution obtained the best results (Figure 6).

Thus, the proposed Hill/L equation preserved and extended the physical content of the classical Hill equation. With the same number of fitting parameters as the Adair equation, the Hill/L equation had a higher approximation ability than the former. Calculating the parameters of this equation, in contrast to Adair’s equation, produced a stable result when optimizing the search for the best approximation parameters [43,44]. It should also be noted that the Hill/G equation had similar properties to the Hill/L equation, except for a weak approximation of the curve in the section where the partial pressure of oxygen did not exceed 3 mm Hg.

### 3.5. Fitting and Derived Parameters in the Modified Hill Equations

The fitting and derived parameters in the Hill/G and Hill/L equations enabled a more complete description of the oxyhemoglobin dissociation curve and an assessment (albeit formal) of the cooperativity of oxygen molecule binding. However, such generally accepted constants as *p*_50_ (*K_h_*), *K_m_*, EC50 and IC50 [45] are convenient, but also formal, estimates of the half-maximum value of the corresponding function. In principle, they could be replaced in the corresponding equations [46,47,48,49] with any constants expressed as a percentage; for example, *p*_95_ (the full saturation tension presented as a constant) or in fractions of 1/*e*, 1/π, etc. [45,46,47,48,49].

The fitting parameter *p*_50_ corresponds with a similar value for the classical Hill equation. The mean value of the Hill coefficient $\bar{h}$ was calculated for all uniformly distributed points of the model curve. From the fitting parameter *h*_max(__−1)_, the maximum value of the Hill coefficient *h*_max_ was calculated using the following formula:

(18) $$h_{\max}=h_{\max(-1)}+1.$$

Using Equation (19), we could estimate the maximum possible relative cooperativity of the subunit interaction [50]:

(19) $$h_{\max}=\theta (n)\left(n-1\right)+1,$$

where *θ*(*n*) is the relative cooperativity coefficient and *n* is the number of oligomer subunits.

The value of the Hill coefficient *h_i_* for any *p_i_* could be found through the variable *h_i_*_(−1)_ similar to *h*_max_ (18):

(20) $$h_{i}=h_{i(-1)}+1.$$

The value of ∆*h*, as the difference between *h*_max_ and $\bar{h}$, provides an idea of the dispersion of cooperativity as a function of the partial pressure of oxygen.

The value of the partial pressure of oxygen, *p*_max_, corresponds with the highest value of the Hill coefficient (*h*_max_) and is determined by the fitting parameter ln *p*_max_. The value of *p*_max_ determines the degree of hemoglobin oxygen saturation HbO_2_, % (for *p_max_*). The difference between the degree of oxygen saturation at *p*_max_ and its 50% saturation is ∆HbO_2_, %, which can also be used when analyzing the oxygen-binding properties of hemoglobin. The difference between *p_max_* and *p*_50_, ∆*p*_max_O_2_, estimates the shift of the maximum cooperativity and requires a physical interpretation.

The fitting parameter *s* (*s_g_* for Hill/G and *s_l_* for Hill/L) makes it possible to obtain *pO_2low_* and *pO_2high_* using the following formulae:

(21) $$p_{O_{2}}{}_{low}=\exp\left[\ln p_{\max}-s_{g}{\left(-\ln\omega \right)}^{1/2}\right],$$

(22) $$p_{O_{2}}{}_{high}=\exp\left[\ln p_{\max}+s_{g}{\left(-\ln\omega \right)}^{1/2}\right],$$

(23) $$p_{O_{2}}{}_{low}=\exp\left[\ln p_{\max}-s_{l}{\left(\omega^{-1}-1\right)}^{1/2}\right],$$

(24) $$p_{O_{2}}{}_{high}=\exp\left[\ln p_{\max}+s_{l}{\left(\omega^{-1}-1\right)}^{1/2}\right],$$

where *pO*_2*low*_ and *pO*_2*high*_ are the lower and upper limits of oxygen partial pressure values, respectively, beyond which *h_i_*_(−1)_ < *ωh*_max(−1)_ and *ω* is the fraction expressed from 0 to 1 (for 0.5, more commonly known as half-width at half-maximum or HWHM).

The range of partial pressure ∆*pO_2_* values within which *h_i_*_(−1)_ < *ωh*_max(−1)_ could be determined by the difference:

(25) $$\Delta p_{O_{2}}=p_{O_{2}}{}_{high}-p_{O_{2}}{}_{low}.$$

### 3.6. Calculation of Fitting and Derived Parameters in the Modified Hill Equations

The values of *p*_50_ and *h* were calculated for the classical Hill equation and were 28.82 mm Hg and 2.52, respectively, according to Winslow et al. [27] and 26.38 mm Hg and 2.65, respectively, according to Severinghaus [28].

The values of the fitting and derived parameters of the Hill/G and Hill/L equations characterizing the oxygenation process for the experimental curves according to Winslow et al. [27] and Severinghaus [28] are presented in Table 3 and Table 4, respectively.

Following on from Table 3 and Table 4, the calculated fitting parameters and their derivatives made it possible to analyze the oxygen-binding properties of this heme protein in more detail.

It should be noted that the values of *p*_50_ calculated according to the Hill/G and Hill/L equations almost completely corresponded with the values of *p*_50_ obtained by means of the classic Hill equation. The mean values of the Hill coefficients of the modified equations were comparable with or somewhat lower than those from the classical equation.

There was a good agreement between the values of parameters 1–5 and 12 within the same data set (Table 3 and Table 4). The group of parameters 6 and 9–11 was more labile and was associated with differences in the exponential and hyperbolic distributions. For the Hill/L equation, the parameter ∆*pO*_2_ was expected to have lower values than for the Hill/G equation because of the more peaked distribution.

It is noteworthy that the position of the cooperativity maximum shifted toward higher values of the partial oxygen pressure for all data sets and approximating models.

Earlier [26], we suggested that this could be due to a certain physiological role of the hemoglobin macromolecule because it is a heterotetramer whose subunits, despite their structural symmetry, possess a functional asymmetry. This was confirmed by the difference in the equal weight constants of oxygen binding for these subunits [51]. The use of a model with a higher approximation capability (Hill/L) relative to the previous one (Hill/G) also strengthened these assumptions.

Moreover, based on the analysis of the parameters of the Hill/G approximating model in our previous work [26], we believed that the functional asymmetry of this macromolecule could not be effectively realized in the case of identical subunits (for example, hemoglobin H, which consists of only four β-subunits) [52].

## 4. Discussion and Conclusions

The Hill equation did not approximate well with the experimental points of the oxygenation curve if the Hill coefficient was equal to the number of oligomer subunits. The best fit with the experimental data was achieved when the Hill coefficient was smaller than the number of interacting monomers. At the same time, the value of this coefficient was non-integer and the model itself correctly described the oxygenation curve only in the range from 20–30 to 70–80% of hemoglobin saturation with oxygen.

The introduction of the parameter “relative coefficient of cooperativity *θ*(*n*)”, which coupled the Hill coefficient and the number of subunits, made it possible to explain the nature of the non-integer value of *h*, but it did not solve the problem of the quality of the experimental data approximations [50].

The oxygenation equation proposed by Adair provided the best results in describing the course of the experimental hemoglobin dissociation curve because it has four fitting parameters in terms of the number of ligand-binding constants. However, this equation does not provide an insight into such important parameters of enzymatic kinetics as protein half-saturation with ligands (*K_m_*, *p*_50_) and the degree of cooperative interactions of subunits (*h*).

We considered oxygenation characterized by *p*_50_ and *h* as a function of oxygen partial pressure. Note that the very idea of such a dependence, in which macromolecule-binding constants are considered to be a function of macromolecule filling with ligands, is not new [25,39,40,41].

The modified Hill’s equation [26] we proposed earlier had four fitting parameters (as with Adair’s equation) and the advantages of the original model; it approximated the experimental data better. However, in the range of values of partial pressure below 3 mm Hg, it was inferior in approximation to the Adair equation although, in general, it is comparable with it.

An analysis of the previously obtained approximating equation based on the Gauss distribution (Hill/G) showed a few limitations in its application. The proposed version of the modified Hill equation based on the Lorentz distribution (Hill/L) showed the best results in the approximation of the experimental data. The fitting parameters of both the Hill/L and Hill/G equations, along with the derived parameters from them, allowed a more complete description of the nature of hemoglobin oxygenation. Thus, it was shown that the maxima of cooperativity (*p*_max_) for the considered sets of experimental data lay around higher partial pressures of oxygen relative to the *p*_50_ value.

In conclusion, the proposed model for hemoglobin oxygenation certainly needs to be improved, both in terms of its physical content and mathematical description, given the fact that it provides a very general description of the oxygenation process. Considering the role of the hemoglobin macromolecule as a generalizing physical model for cooperativity in biophysical studies, the proposed equation for oxygenation could be considered in a wider context from tasks of enzymology and pharmacology [36,53] to the modeling of gene transcription regulation [54,55] as well as various dose–response relationships [56,57]; i.e., where the classical Hill equation is already traditionally applied. At the same time, starting from the classical models of MWC [58] and KNF [59], evolving to modern models of cooperative ligand binding [60], repeated attempts have been made to improve these models in order to fully and most accurately match the experimental results [61,62,63,64]. In our forthcoming work, we aim to describe the main mechanisms of the cooperative binding of hemoglobin with oxygen as well as to establish a connection between the different models.

## Figures and Tables

**Table 1:** Approximating functions for the ODC, the coefficient of determination *r*^2^, which was calculated from the experimental data of Winslow et al. [27].

Equation	*r* ^2^
Hill/L	0.999962
Adair	0.999953
Hill/G	0.999944
Hill classic	0.999603

**Table 2:** Approximating functions for the ODC, the coefficient of determination *r*^2^, which was calculated from the experimental data of Severinghaus [28].

Equation	*r* ^2^
Hill/L	0.999951
Hill/G	0.999943
Adair	0.999907
Hill classic	0.999446

**Table 3:** Values of fitting parameters and their derivatives from the Hill equation with various modifications (from the experimental data of Winslow et al. [27]).

No.	Equation Parameters	Hill/G Equation	Hill/L Equation
1	*p*_50_, mm Hg	29.08	29.11
2	$\bar{h}$	2.30	2.30
3	*h*_max(−1)_	1.66	1.68
4	*h*_max_ (*h*_max(−1)_ + 1)	2.66	2.68
5	∆*h* (*h*_max_ − *$\bar{h}$*)	0.36	0.38
6	*p*_max_, mm Hg	52.92	47.82
7	*s_g_*	3.33	—
8	*s_l_*	—	1.80
9	*pO_2low_* (for ω = 0.99), mm Hg	37.92	36.36
10	*pO_2high_* (for ω = 0.99), mm Hg	73.87	62.89
11	∆*pO_2_* (*pO_2high_ − pO_2low_*), mm Hg	35.95	26.53
12	HbO_2_, % (for *p*_max_)	83.12	79.09

**Table 4:** Values of fitting parameters and their derivatives from the Hill equation with various modifications (from the experimental data of Severinghaus [28]).

No.	Equation Parameters	Hill/G Equation	Hill/L Equation
1	*p*_50_, mm Hg	26.82	26.86
2	$\bar{h}$	2.62	2.60
3	*h*_max(−1)_	1.82	1.82
4	*h*_max_ (*h*_max(−1)_ + 1)	2.82	2.82
5	∆*h (h*_max_ − *$\bar{h}$*)	0.20	0.22
6	*p*_max_, mm Hg	80.85	67.28
7	*s_g_*	3.59	—
8	*s_l_*	—	1.91
9	*pO_2low_* (for ω = 0.99), mm Hg	56.42	50.25
10	*pO_2high_* (for ω = 0.99), mm Hg	115.85	90.08
11	∆*pO_2_* (*pO_2high_ − pO_2low_*), mm Hg	59.43	39.83
12	HbO_2_, % (for *p_max_*)	95.74	93.04

## Data Availability

Not applicable.
